# Pelotherapy, thalassotherapy, and electrotherapy for skin treatment: current insights and future perspectives on electropelotherapy

**DOI:** 10.1007/s00484-025-02955-y

**Published:** 2025-06-18

**Authors:** Lara Almeida, Fernando Rocha, Carla Candeias

**Affiliations:** GeoBioTec Research Unit, Geosciences Department, University of Aveiro , Campus de Santiago, Aveiro, 3810-193 Portugal

**Keywords:** Electrotherapy, Clays, Thalassotherapy, Salts

## Abstract

This work aims to review and summarize the existing knowledge on Electropelotherapy, a new therapeutical approach for physical rehabilitation practices. Research was conducted on scientific literature covering general topics (e.g., clays, peloids) and the positive health outcomes. More specific keywords (e.g., thalassotherapy, the role of Dead Sea salts, electropelotherapy, dermal bioaccessibility), focusing on improving human healthcare and well-being. A growing global interest for natural muds is linked to its documented health and well-being benefits. Thalassotherapy is a traditional therapeutical technique extensively explored and applied over the years. Similarly, electrotherapy is a well-established method for physical rehabilitation. It is proven that both therapies can provide positive benefits for human health. Studies showing the success of combining both therapies are reduced, with only one pilot study conducted on equines, to assess the potential effectiveness. Additional studies are crucial to fully understand the potential and clinical applications of electropelotherapy in physical rehabilitation, and potentially contributing to the development of innovative strategies to enhance patient recovery.

## Introduction

Focusing on human health, the use of clay minerals dates back to the earliest period of humankind (Carretero 2002), with clay-based peloids, the most commonly used in Mediterranean countries (Pozo et al. 2013). Over the years, different therapeutical techniques have been explored. Electrotherapy, recognized as a medical treatment (Tiktinsky et al. 2010), is a fundamental methods in physiotherapy practice (Watson 2000). According to Tiktinsky et al. (2010), this therapy is effective in producing different physiological effects, such as reducing acute and chronic edema, promoting tissue repair, pain relief. Pelotherapy involves the topical application of muds (usually clay-based) (Bastos et al. 2022), with several studies demonstrating its positive benefits in dermal and rheumatological applications. Thalassotherapy is a specialized form of pelotherapy and climatotherapy, that uses products directly from the sea, e.g., seawater, mud, sand, and algae, combined with the therapeutic benefits of the climate (Kazandjieva et al. 2008). Proksch et al. (2005) suggested that Dead Sea salts contribute significantly to human health and well-being, showing positive effects on inflammatory diseases. According to Anderson and Meade (2014), skin is a highly complex organ, the largest on the human body, that functions as a protective barrier, with the stratum corneum playing a crucial role. Dermal bioaccessibility, relies on the ability to penetrate this barrier and reach the underlying active epidermis (Carlos et al. 2023).

The combined application of electrotherapy and pelotherapy, and/or thalassotherapy, remains limited, with no conclusive evidence supporting its potential in skin treatments or the ability to effectively penetrate the skin barrier, including the stratum corneum. The present work aims to explore the potential of combining these two non-invasive techniques, electrotherapy and geomaterial-derived peloids, in physical rehabilitation, particularly in humans, for skin recovery, based on the limited available evidence. By synthesizing the available evidence, this review seeks to highlight the potential benefits, challenges, and areas for further research in this emerging therapeutical approach.

## Medical context

In medicine there is a long tendency to define health as the ability of ideal function (Ferrans et al. 2005). Over the years, positive health has been regarded as “more than the absence of illness” (WHO, 1946). Nearly 80 years ago, the World Health Organization (WHO, 1946) defined health, not just as the absence of diseases or infirmities, but a state of complete mental, physical and social well-being, on which all human beings, regardless of race, religion, political belief, economic or social status, have the fundamental right to the highest attainable standard of health conditions, in line with the United Nations (UN) Sustainable Development Goals (SDG) n.º 3 to “Ensure access to quality health and promote well-being for all at all ages”. WHO, and the Copernican revolution, disseminated this objective over the world, having an important role in the development of national health care systems, pushing countries beyond the traditional boundaries of health limited to individuals physical conditions (Leonardi 2018). Gostin and Taylor (2008) expressed health treatments as “a field that encompasses the legal norms, processes, and institutions needed to create the conditions for people through the world to attain the highest possible level of physical and mental health”. Over the last 50 years, health care has increasingly focused on assessing life quality (Ferrans et al. 2005). This term is used to refer a variety of different conditions, e.g., health status, life satisfaction, happiness, physical functioning, psychosocial adjustment, symptoms, and well-being (Ferrans et al. 2005). Viseras et al. (2007) proposed that healthcare products promoting health and well-being should include several categories. These include medicinal products for disease treatment, cosmetic formulations, perfumes, and materials designed to correct body odors and imperfections. Additionally, products should protect or maintain the external parts of the body. Functional foods, which are special nourishment designed to be consumed as part of a diet, also fall under this category. These foods contain biologically active components that may enhance health or reduce disease risk. Different medicinal and cosmetic products have been formulated to be in direct contact with external parts of the human body, e.g., skin, hair, nails, lips, or with the mucous membranes of the oral cavity and teeth structure (Viseras et al. 2007).

## Clays, clay minerals and human health

### Historical background

Clay minerals have been used for healing purposes since prehistoric times, being its use as old as humankind (Carretero 2002; Gomes and Silva 2007). There are historical references of the use of mud/clays, known as “medicinal earths” in Mesopotamia, Ancient Egypt and Ancient Greece, stating the healing power on wounds and soothing skin rashes (Gomes and Silva 2007). In general, clay minerals are considered essential to life, and to human health in particular (Gomes and Silva 2007). These materials have been included in different health care formulations (Viseras et al. 2007), being used for therapeutic purposes, given its low-cost and the low difficulty involved in its synthesis processes, being commonly applied in SPAs treatments, aesthetic medicine practices, and as active principles, or excipients, in pharmaceutical formulations (Carretero 2002).

### Clays and clay minerals

Clays and clay minerals are found in a relatively limited range of geologic environments, including continental and marine sediments, soil horizons, volcanic deposits, thermal fields, and weathering rock formations (Al-Ani and Sarapää 2008). It may also originate from low-temperature metamorphic processes or hydrothermal reactions in the upper crust, and precipitation and accumulation in soils and sediments to progressive diagenetic processes (Warr 2022). Most clays result from the interaction between aqueous solutions and rocks, with the dissolution and recrystallization processes at this interface, leading to the formation and transformation of clay minerals (Velde 1995). Additionally, clay minerals occur in specific types of geological materials, such as, sediments and sedimentary rocks, being common in hydrothermal deposits (Valaskova and Martynková 2012). Thus, the cycle of clay starts with the formation of clay minerals and their accumulation in soils through weathering of primary rocks (Al-Ani and Sarapää 2008). Once formed, play a catalytic role in biogeochemical cycles within the critical zone, where life interacts with the geosphere, hydrosphere and atmosphere once formed (Schroeder 2018).

The definition of ‘clay’ was first formalized in 1546, and has been revised numerous times since then, considering, among others, plasticity, particle size, and hardening on firing (Kumari and Mohan 2021). Guggenheim and Martin (1995), proposed the definition for clay as ”a naturally occurring material composed primarily of fine-grained minerals, which is generally plastic at appropriate water contents and will harden when dried or fired”. The term “clay mineral” refers to phyllosilicate minerals and others that impart plasticity to clays, and harden upon drying or firing (Guggenheim and Martin., 1995; Al-Ani and Sarapää 2008). Based on their occurance, clays can be categorized as residual or sedimentary, if at the place of origin and formed through surface weathering, or if transported from the original location by erosion and deposited in a new and potentially distant area, respectively (Kumari and Mohan 2021). The composition of clays is influenced by the mineralogical and chemical composition of the source material (Mana et al. 2017). In turn, physicochemical and chemical properties are determined by structure and composition, and can be influenced by ionic charge variations that alter the most common structural arrangement (Al-Ani and Sarapää 2008). Clays and clay minerals are associated with some specific properties, e.g., cation exchange capacity (CEC), adsorption, surface charge, and swelling (Schoonheydt and Johnston 2011).

Each clay mineral has a unique structure, being crucial in defining its specific application (López-Galindo et al. 2007). The structure of clay particles consists of layers, each formed by the combination of tetrahedral (T; SiO_4_) and octahedral (O; Al_2_O_3_) sheets, linked by oxygen atoms, with the unshared one appearing as hydroxyls (Nascimento 2021). The fusion of two sheets creates a layer, and multiple layers can be bonded in a clay crystallite through interlayer cations, Van der Waals forces, electrostatic forces, or hydrogen bonding (Uddin 2008). Additionally, the arrangement of tetrahedral and octahedral sheets accounts for the diversity of clay minerals groups: kaolinite (1:1); montmorillonite or smectite, illite and vermiculite (2:1), and chlorite (2:1:1). In these groups of minerals, each layer consists of: 1:1 - one tetrahedral sheet is bonded to one octahedral sheet; 2:1 - one octahedral sheet is sandwiched between two tetrahedral sheets; and 2:1 - an additional octahedral sheet is adjacent to the 2:1 layer (Meunier 2005; Ovincy et al. 2024). Clays particle size is an important feature but not consensual: 2 μm for geologists and soil scientists, 4–5 μm for sedimentologists, and 1 μm for colloidal chemists (Guggenheim and Martin 1995; Kumari and Mohan 2021).

### Healing properties of clay minerals

Natural clay has been used historically for different purposes, including therapeutic approaches, due to its healing capacity (Ovincy et al. 2024). Clays healing capability is known since ancient times. There are records that early Mesopotamians used clay to treat wounds and stop bleeding (Ovincy et al. 2024). According to Gomes and Silva (2007) “man, and minerals (here considered as natural inorganic solids, generally crystalline) are chemical systems having in common, in their composition, the major chemical elements oxygen, hydrogen, carbon, nitrogen, the so-called mineral salts sulfur, phosphorous, sodium, potassium, magnesium, and some others called oligoelements or micronutrients or trace minerals such as Fe, Cu, Zn, Se, Mn, I, F which are essential both to life and to the formation of minerals”.

The use of clay minerals for any specific application depends firstly on its structure (Viseras et al. 2007). There are various types of clay that are often used for medical and pharmaceutical purposes and therapies, e.g., kaolinite, montmorillonite, talc and halloysite, with kaolinite, illite, halloysite, montmorillonite and other smectites being the five common types of clay minerals (Ovincy et al. 2024). Specifically, smectites, palygorskite, kaolinite and talc are the clay minerals used in pharmaceutic fields, while smectites and kaolinite are the most common in SPAs (illite and palygorskite can also be applied) (Carretero 2002).

Clay minerals can extend their applications to the field of aesthetic medicine, where they are used in the formulation of cosmetic products (Carretero 2002), with guides and protection of the European Community Directive 76/768/ECC (López-Galindo et al. 2007). The efficiency in the pharmacological and cosmetic function, abrasives, absorbents and adsorbents, lubricants, glidants, anticaking and coating agents, and emulsion stabilizers are some practical products (López-Galindo et al. 2007). There are several studies showing the efficiency of clays in health applications (e.g., Carretero 2002; Carretero et al. 2006; Gomes and Silva 2007; Viseras et al. 2007; Williams and Hillier 2014). Recently, Ovincy et al. (2024) concluded that clay minerals are effective in the treatment of injuries due to their anti-inflammatory and antibacterial properties, promoting fibroblast regeneration and circulation.

Clays and clay minerals can enter the human body through inhalation, ingestion or dermal absorption (Finkelman 2019), as well as exposure to potentially toxic heavy metals associated, e.g., arsenic, cadmium, lead, mercury (Gomes et al. 2021). While uncommon, there are circumstances when clays can pose a risk to human health, e.g., if clay particles are persistently inhaled, leading to respiratory disorders; geophagy (voluntary daily ingestion); or absorbed through the skin, being the severity of the potential resulting diseases dependent on the dose and duration of exposure. Nevertheless, according to Carretero (2002), some special clays and clay minerals are used both in therapeutical treatments externally (topical, i.e., pelotherapy and thalassotherapy) and internally (oral).

## Peloids and pelotherapy treatments

### Historical background

**Fig. 1:**
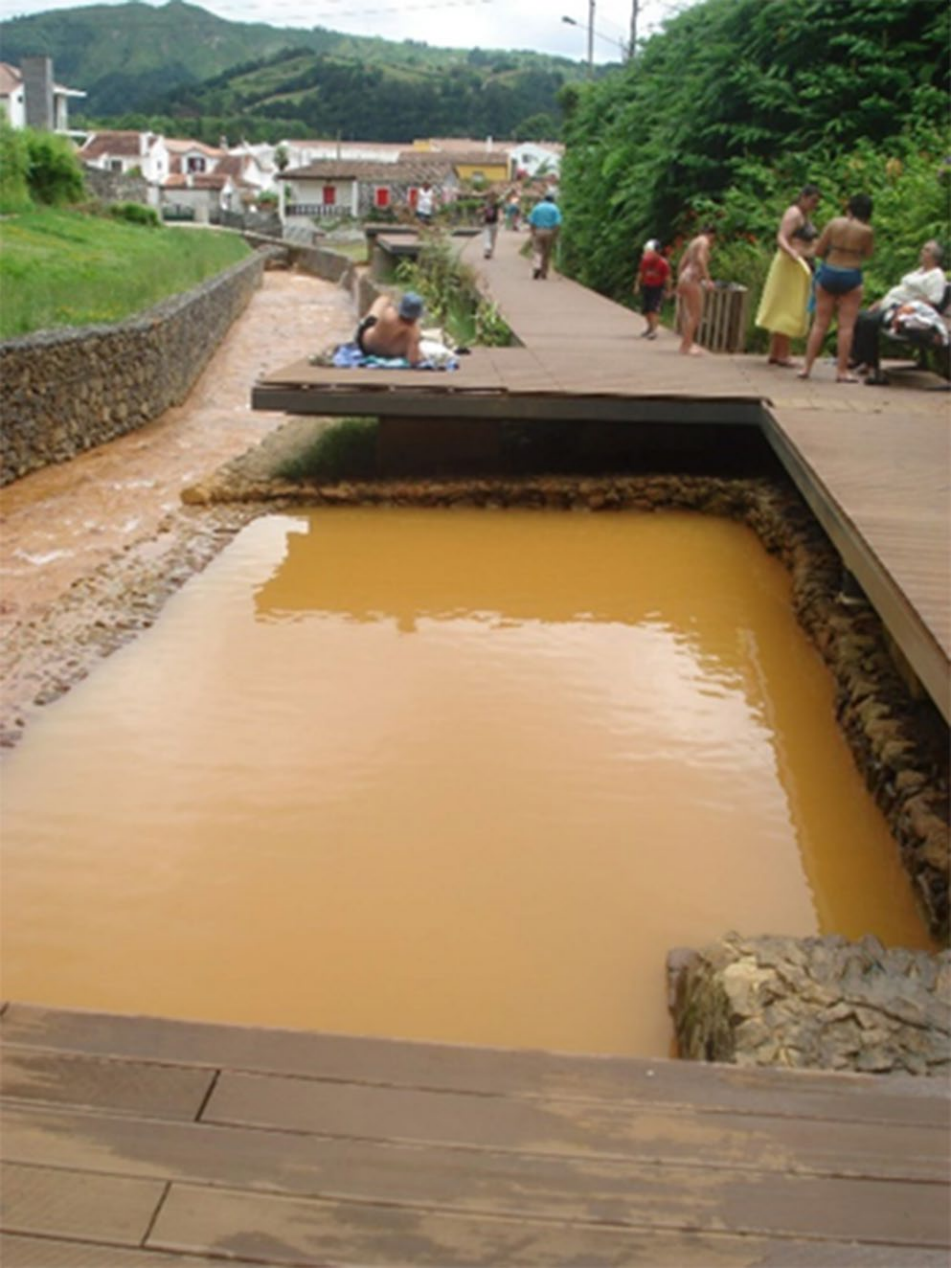
Mud baths (Poça da Dona Beija, S. Miguel, Azores, Portugal; Bastos et al. 2022)

Peloids have been used since ancient times as therapeutic agents that provide heat (Fig. 1), and pelotherapy remains a key practice in health resort medicine, used in the form of balneotherapy and thalassotherapy (Maraver et al. 2021).

The use of peloids, a mixture of natural materials with healing properties with therapeutic and cosmetic purposes, have been studied by numerous scientists over the years, being characterized by its composition and healing potential, in particular for dermal applications (Quintela et al. 2012; Potpara et al. 2017; Bergamaschi et al. 2020; Katona et al. 2020; Almeida et al. 2023a), but also with rheumatological purposes (Codish et al. 2005; Evcik et al. 2007; Fioravanti et al. 2007; Fraioli et al. 2011). According to Maraver et al. (2021) since ancient times, when heated, peloids have been used as an healing aid in specific diseases, such as musculoskeletal disorders of the knee (Gálvez et al. 2019), spine (Cozzi et al. 2020), hand (Tenti et al. 2020), and fibromyalgia syndrome (Bağdatlı et al. 2015). Pozo et al. (2013), reported that peloids, with a clay base, were the most commonly used in Mediterranean countries (e.g., France, Greece, Italy, Turkey, Dead Sea area), whilst peat based peloids were preferred in Northern and Central Europe (e.g., Czech Republic, Germany, Hungary), and peloids involving sulphur-rich compounds were used in other parts of the world, such as Argentina.

Since ancient times, peloids have been used regularly, as thermal agents in SPAs, health resorts and medical centers (Carretero et al. 2006). Currently, balneotherapy and thalassotherapy are the main therapies using peloids (Maraver et al. 2021). Balneotherapy, from the latin *balneum* (bath), refers to the medical use of water, classically used for bathing in thermal or mineral waters, usually combined with other treatments, e.g., hydrotherapy, physical exercise and mud packs (Munteanu and Munteanu 2019; Nasermoaddeli and Kagamimori 2005). Thalassotherapy, derives from the Greek word *thálassa*, related to sea or ocean, a therapeutic technique that includes seawater-based treatments (Munteanu and Munteanu 2019).

### Definition of peloids

According to Gomes et al. (2013), the term peloid and the International Classification of Peloids were approved by the General Assembly of the International Society of Medical Hydrology (ISMH), during the “IVème Conférence Scientifique Internationale” held in Dax (France) in October 1949. Over the years, different definitions have been formulated for the concept of peloid. Lewis (1933) defined peloid as “any natural product constituted of a uniform mixture of finely divided organic and inorganic matter with water, prepared and applicable in medical practice as cataplasm for external treatment”, Massy et al. (1949), defined it as “natural medicines, the result of both geologic or biologic processes, which being presented in a state of fine division mixed with mineral water are utilized in baths or in wraps”, Pisani (1951), defined it as “hyperthermal or hyperthermalized therapeutic means derived from the intimate primary or secondary mixture of a solid component constituted of a natural geologic or phytologic product with a liquid component represented by healing thermal or saline water, and used under the form of pack or bath”, and Porlezza (1965), defined it as “finely grained inorganic or organic substances originated through geologic processes than in nature could be presented either dry or mixed with water, and finding application in the medical practice under form of bath or pack”. Gomes et al. (2013) proposed a new and most recent definition of peloids as “a mature mud or mud suspension or dispersion with curative or cosmetic properties, consisting of a complex mixture of fine-grained materials of geological and/or biological origin, mineral or sea water, and organic compounds commonly arising from some biological metabolic activity”. Particularly interesting, for Gomes et al. (2013) peloids are medical peloids “when their therapeutic properties were recognized by the national authorities who approve drugs based upon medically assisted epidemiological studies carried out by physicians specialized in medical hydrology and physiotherapy”, and cosmetic peloids “when they have specific cosmetic properties which were recognized by the laboratories specialized and certified in dermocosmetics”.

### Classification of peloids

**Fig. 2:**
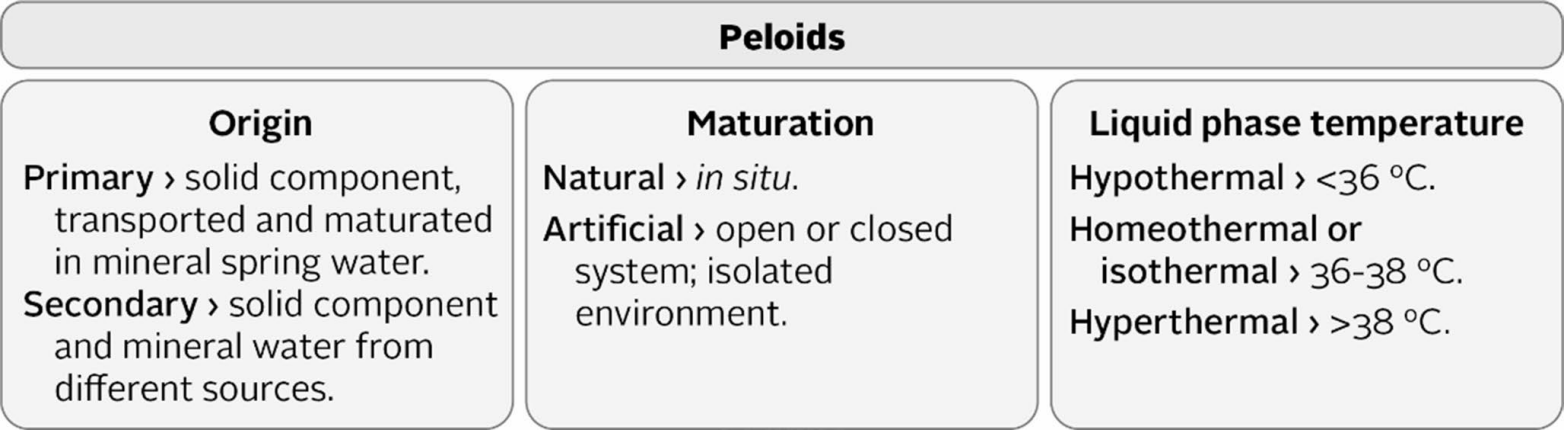
Peloids classification considering the origin, maturation, and liquid phase temperature processes (adapt. Gomes et al. 2013)

Similarly to the definition of peloids, there were different classifications of peloids to this day (Fig. 2). The International Classification of Peloids adopted by ISMH, incorporated the temperatures of the liquid phase and the maturation process. The liquid phase, in which sea water was considered for the first time as component of the liquid phase, was classified as hyperthermal (> 38 °C), homeothermal or isothermal (36–38 °C), and hypothermal (< 36 °C). Nasermoaddeli and Kagamimori (2005), described mineral water temperature as being cold (< 20 °C), hypothermal (20–30 °C), thermal (> 30–40 °C), or hyperthermal (> 40 °C). The maturation process was mentioned as natural, at the occurrence site, or artificial, when in open or close system, isolated from the air (Gomes et al. 2013). Veniale (1998), classified the peloids based on the origin, as primary and secondary peloids. A peloid is considered a primary or secondary peloid when, the solid component has been mechanically transported as particulate dispersed material and deposited in the mineral water of the spring, or the solid component and the mineral water come from different sources, respectively. Other classifications have been proposed by Armijo et al. (2005); Legido et al. (2007), and Lüttig (2004). According to Armijo et al. (2005) and Legido et al. (2007), as discussed during the 3rd Symposium on Thermal Mud held in Dax (2004), two primary categories of peloids can be identified: (i) extemporaneous or ad hoc peloids, which consist of muds or clays simply mixed with mineral water without undergoing any maturation process, and (ii) matured peloids, which involve muds or clays combined with mineral waters and subjected to natural or artificial maturation. The classification of peloids proposed by Lüttig (2004), originally developed in 1990 and recommended to the International Peat Society for international use, is primarily based on geological parameters. This system identifies three main categories of peloids: eupeloids, which refer to natural, unprocessed materials; parapeloids, which are physically modified through crushing, milling, or surface treatment, and peloid apogones, defined as artificially produced peloidic substances created by adding liquid carriers, such as mineral or thermal waters. Gomes et al. (2013), proposed a classification of peloids taking into account the origin, as peloid in *sensu strictu* (maturation in tanks with mineral water) and natural peloid, composition (inorganic, organic or mixed peloid), and application (medical or cosmetic peloid).

### Peloids application

The period of peloids application of 20 to 30 min, was accepted by several authors (Carretero et al. 2006; Gomes 2018; Tateo and Summa 2007; Veniale et al. 2007). Depending on the SPA and the illness or part of the body to be treated, there are different ways of applying the peloids. They are usually applied in the form of a poultices on a certain area of the body undergoing treatment (e.g., back, joints, arms). After, the area being treated is covered with a waterproof material and a blanket or sheet, to prevent water, contained in the peloid, evaporation and to allow heat dissipation. There are other types of peloids application, such as, (a) with a brush all over the body and left to dry while it exerts its therapeutic action, then placed in a bath of mineral-medicinal water to help remove the dried peloid from the skin; (b) only a part of body (e.g., feets or hands) is immersed in a bath with peloid; and (c) complete immersion in a bath containing the peloid diluted in mineral-medicinal water. It is common to perform body massages, when with the peloids in the form of a mud suspension instead of oils (Carretero 2020a). According to Carretero (2020a), in general, healing peloids are applied with a temperature of 40 to 45 °C, to combine heat therapeutic properties allied to those of the peloid itself.

### Peloids maturation

**Fig. 3:**
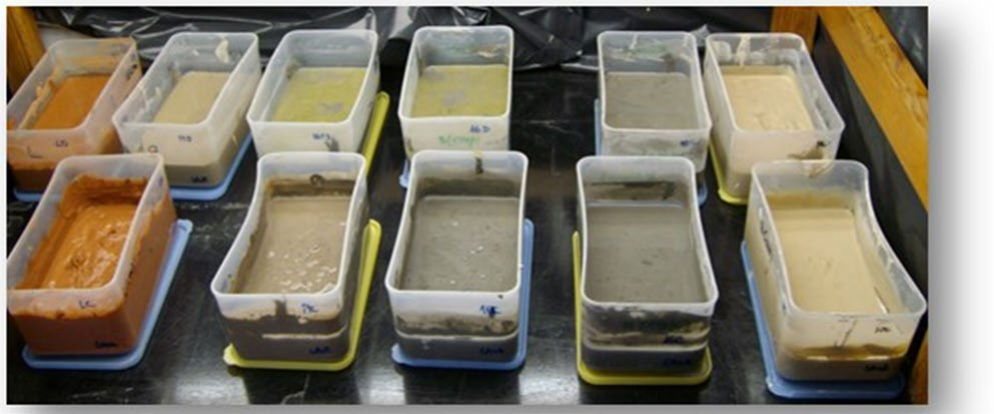
Peloid maturation

According to Gomes et al. (2013), maturation can occur naturally, at the geological site of origin, or artificially. This process is essential to improve and stabilize the therapeutic properties of the peloids (Sánchez et al. 2018), and can vary significantly in duration, ranging from 60 days to over 2 years (Veniale et al. 2004). Carretero (2020a) suggested that maturation period usually varies between a few days, months or even years. The artificial process consist of a mixture of a solid phase (typically virgin clays) with mineral water, such as seawater or mineral-medicinal waters (Fig. 3) (Veniale et al. 2004), where the interaction between the two phases promotes mineralogical, chemical, and physical alterations (Carretero 2020a). Microorganisms can develop during maturation (Quintela et al. 2013, 2015), depending on the nature of the solid and minero-medicinal water, and the circumstances in which the process takes place. It is important to refer that the maturation product, named peloid, combines characteristics from both solid and liquid phases, influenced by the temperature at which maturation occurs, typically aligned to the natural temperature of the spa minero-medicinal water source (Carretero 2020a).

Over the years, the formulation of peloids with different types of water has been studied (Veniale et al. 2004; Carretero et al. 2007; Gámiz et al. 2009; Rebelo et al. 2015; Bastos and Rocha 2023). However, there is limited research specifically focusing on the maturation of peloids with added salts.

### Properties of peloids

Peloids are widely recognized for its application in thermotherapy treatments (Carretero 2020a), due to the biological effects, metabolic and enzymatic activity, vascular, neuromuscular, analgesic, and modifications of the viscoelastic properties of the tissues (Maraver et al. 2021). The anti-inflammatory and immunological actions have also been reported (Gálvez et al. 2020).

The solid phase of the peloid is essential as it acts as a vehicle, allowing the sustained release of heat and enhancing the effectiveness of the therapy (Maraver et al. 2021). According to Maraver et al. (2021), in order to be considered peloids, material must be easy to handle and offer a pleasant sensation when applied in the skin, as well as having a low cooling rate. Carretero et al. (2006) added a high absorption and cation exchange capacities and good adhesiveness.

Carretero (2020a) carried out a review of specific properties, based on literature related to the use of peloids. Mineralogically, peloids are usually composed of phyllosilicates (i.e., smectites, kaolinite, illite and chlorite), quartz, calcite, feldspars and dolomite, and in minor amounts, gypsum, halite, aragonite and zeolites. The chemical composition is directly related to the mineralogical composition of the solid and water-based formulation, with the major (Si, Al, Fe, Ca, Mg, Na, K) and minor (Ti, Mn) elements present in the peloids. Particle size, specific surface, plasticity, swelling power and index, abrasiveness, density, viscosity, water content, pH, cation exchange capacity (CEC) and exchangeable cations, specific heat capacity, thermal conductivity, diffusivity, and retentivity, are the physicochemical properties assessed by researchers working with peloids for therapeutic purposes (Quintela et al. 2012; Rebelo et al. 2011). Almeida et al. (2023b), explored the technological properties of clay sediments for potential use in pelotherapy. An exchange between the ions of the liquid phase and the interchangeable ions of the solid phase (phyllosilicates) is possible with high CEC (Carretero 2020a), that contributes to the modifications of other properties, e.g., swelling index, water retention, viscosity, adhesiveness (Veniale et al. 2007). Considering that peloids are used in thermotherapeutic applications, the thermal properties are crucial. The effectiveness increases with high specific heat and low thermal conductivity, as the heat is retained more efficiently, ensuring a consistent and elevated temperature throughout the application session (Carretero 2020a). The biological fraction of the peloids, comprises microbiota found in the two phases, liquid (mineral-medicinal waters) and solid (clays, peat, or sediments), and may include microorganisms that proliferate during the maturation process (Maraver et al. 2015). The different biologically active compounds with therapeutic effects and actions are formed during maturation, specifically microalgae and cyanobacteria found in peloids (Quintela et al. 2013; Carretero 2020b). Recent studies characterized the biological fraction of peloids (e.g., Quintela et al. 2015; Centini et al. 2020; Zampieri et al. 2020; Demay et al. 2021).

## Thalassotherapy

The aim of society towards a culture of leisure and health has led to an increase in demand for health tourism. Thalassotherapy emerged in the middle of the 18 th century (Kazandjieva et al. 2008) and is derived from the Greek terms “thalassa” (sea or ocean), and “therapy” (Antonelli and Donelli 2025). Kazandjieva et al. (2008) described it as “a modality of therapeutic and prophylactic application of sea water, mud, algae, sand and climate”. Thalassotherapy is defined in ISO 17,680/2015 as a treatment that involves the use of seawater and substances directly extracted from the sea environment, administered at a marine site under medical supervision. This treatment is both therapeutic and preventive, promoting wellbeing and healthcare by utilizing marine elements such as seawater, seaweed, marine mud, sands, and other substances derived from the sea environment. Thalassotherapy is a special method of climatotherapy, resulting from the combination of sea and climate cures, where natural resources are important elements. Sea water, and its products, have been used, for thousands of years, due to the curative properties. According to Lucchetta et al. (2007), “Throughout the ages, the interest in the use of sea water in medicine has fluctuated from century to century and from nation to nation”. Sea water was one of the most widely used therapeutic agents during the ancient Greek and Roman ages (Kazandjieva et al. 2008). In thalassotherapy, sea water is used and characterized by its own properties (Munteanu and Munteanu 2019). This type of water is particularly known for its high mineral content, high density, and chemical composition, which is rich in chlorides, primarly sodium and magnesium, as well as others e.g., calcium, potassium, and iodine (Munteanu and Munteanu 2019).

Thalassotherapy has been the subject of several studies in different fields, e.g., rheumatology (Andrade et al. 2008; Zijlstra et al. 2005), recovery of muscle damages (Kim et al. 2020), skin diseases, such as psoriasis (Kazandjieva et al. 2008), atopic dermatitis, vitiligo and other eczemas (Riyaz and Arakkal 2010), and recuperation of skin and well-being after cancer therapies (Mourelle et al. 2023).

### Dead sea

Dead Sea (DS), the world’s deepest Salt Lake, is recognized as the largest natural saline reserve in the world, renowned for its healing and cosmetic benefits (Bawab et al. 2018). It lies in the East Syrian rift valley, surrounded by the Moab Mountains to the east and the Judean Mountains to the west, making it one of the most hypersaline waterbodies on Earth (Oren 2010). The lake covers an area of ~ 630 km^2^, with a maximum depth up to 300 m (Oren 2010), being unique for its combination of natural resources, unequaled globally (Moses et al. 2006). Resources include sunlight, which is weakened due to the long distance to reach DS basin, of 415 m below sea level, and the scattering caused by a persistent fog that covers the sea for most of the year (Moses et al. 2006). With a pH of ~ 6, and salt content of ~ 348 g L^−1^, its salinity is ~ 10 times higher than in oceans (Oren 2010). The DS atmosphere contains 10% more oxygen when compared to other seas, possibly attributed to its exceptionally low altitude (Bawab et al. 2018). Water usually contains ~ 348 of mineral salts per liter, being Mg 1.98 mol L^−1^, Na 1.54 mol L^−1^, Ca 0.47 mol L^−1^, K 0.21 mol L^−1^, and Cl 6.48 mol L^−1^ and Br 0.08 mol L^−1^, the main cations and anions, respectively (Oren 2010). However, the salinity is not the only extraordinary feature of this environment, it also has natural thermo-mineral waters, mineral muds, high levels of Br in the air, and a high Se content in the local drinking water (Halevy and Sukenik 1998).

In the Bible, DS was referred as a “salt area”, by Greeks “asphaltic area”, and by Arabs “sea of Araba”, being considered an important therapeutical area (Riyaz and Arakkal 2010). According to Riyaz and Arakkal (2010), Aristotle was the first to report DS therapeutic importance, but was the French chemist Lavoisier who was the first researcher to study DS products in the 18 th century. Over the years, several studies have been carried out using DS materials, promoting health and well-being. Scientific reports highlighted DS as an attractive destination for patients who seek therapy for various skin diseases and rheumatic disorders. Katz et al. (2012) revealed scientific evidence of its therapeutical effects in the treatment of different diseases, such as dermatological and rheumatological. The use of these specific environment has been strongly studied for skin diseases, particularly psoriasis (Halevy et al. 1997; Abels and Kipnis 1998; Harari et al. 2011; Kopel et al. 2013; Emmanuel et al. 2020), even in cases of pediatric-onset (Ben-Amitai and David 2009), and atopic dermatitis (Halevy and Sukenik 1998). Other authors have explored its impact on cosmetic effects, including anti-aging and skin rejuvenation (Yan et al. 2024), musculoskeletal disorders, namely fibromyalgia (Buskila et al. 2001) and osteoarthritis (Sukenik et al. 1990, 1999; Sherman et al. 2009), cardiovascular issues, such as chronic heart failure (Moses 2012), and pulmonary diseases, such as cystic fibrosis and chronic obstructive pulmonary disease (Moses 2012).

### Salts and human health

Magnesium is the fourth most common element in the human body, following Ca, Na, and K (Polefka et al. 2012), being the second most common intracellular cation (Schwalfenberg and Genuis 2017). Over 60% of the Mg present in the human body is found in the skeleton, and remainder is distributed within cells, where it plays a crucial role in energy metabolism and cell replication (Polefka et al. 2012). There is an increasing support for the use of Mg supplements through multiple health areas, e.g., cardiac arrythmias, diabetes and its complications, premenstrual syndrome, hyperlipidemia and asthma, helping with depression, attention deficit disorder, cataract prevention, smoking cessation (Schwalfenberg and Genuis 2017). Denda et al. (1999), confirmed that Mg salts accelerate the barrier repair.

Dead see Mg content is ~ 28 times higher than the Mediterranean Sea (Sudan 2024). Oren (2010), described Mg as the most abundant cation present in DS water, being known to exhibit favorable effects in inflammatory diseases (Proksch et al. 2005). The high salinity and presence of Mg and others elements play an important role in human health and well-being. Manoharan and Kaliaperumal (2021) showed the ability of these ions to penetrate the skin layers and the enhance immunity that ultimately help to relieve psoriasis. Proksch et al. (2005), emphasized the effectiveness of baths with a MgCl-rich salt in treating atopic dermatitis, specifically by improving skin barrier function, enhancing hydration of the stratum corneum, and reducing skin inflammation. A recent study highlighted the significant potential of Mg in DS salts for treating atopic dermatitis, owing to its specific properties, such as high concentration, distinct composition, and anti-inflammatory effects (Sudan 2024).

## Electrotherapy and pelotherapy: a combined approach

**Fig. 4:**
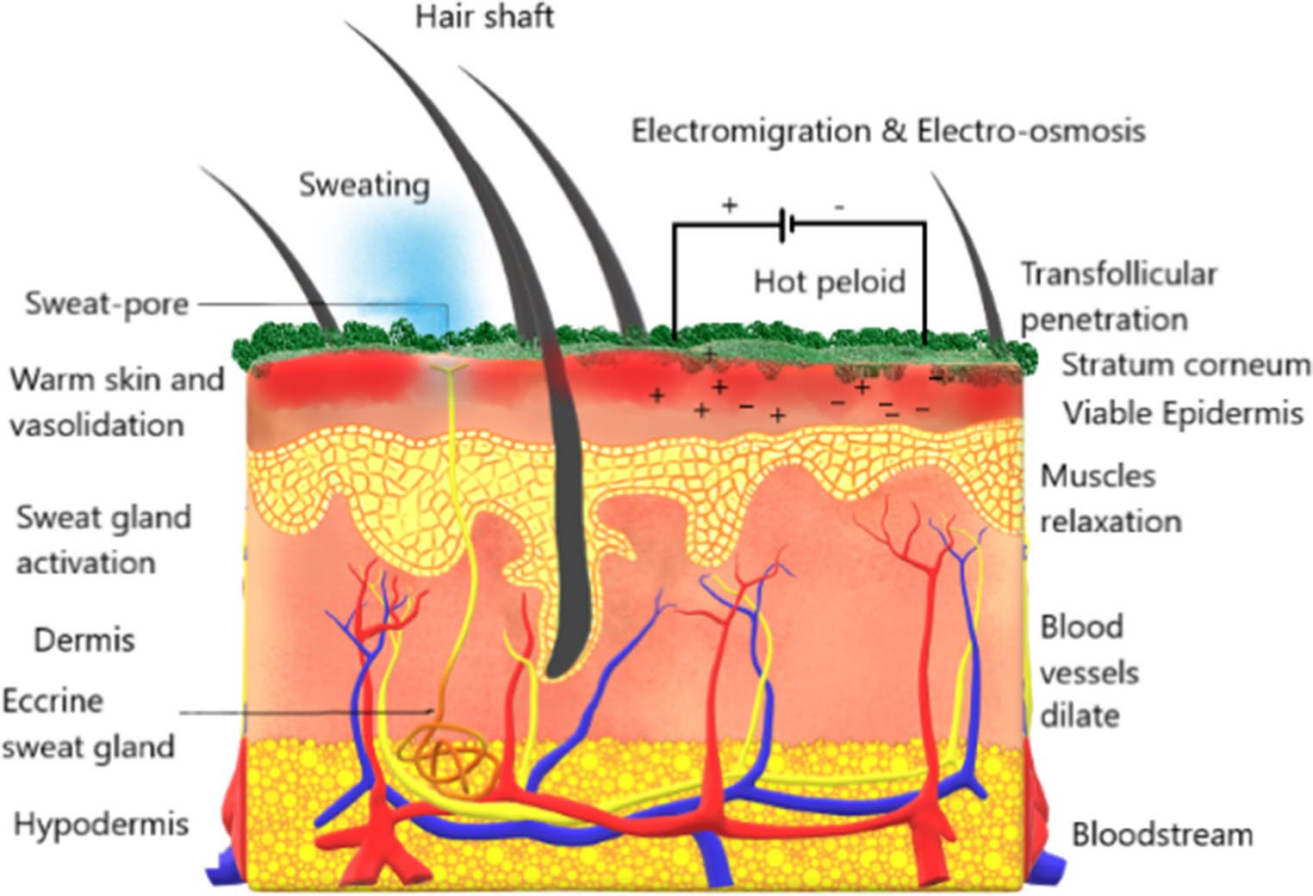
Routes of penetration by electric current and heat, aiming iontophoresis in conjunction with pelotherapy (Bastos et al. 2022)

Electrotherapy has been used since the early professional days, as it is one of the fundamental method of the physiotherapy practice (Watson 2000). Electrotherapy or electromagnetic therapy, the use of electrical energy to the body for medical and rehabilitative purposes, is considered a medical treatment (Tiktinsky et al. 2010). This approach comprises the treatment of a range of medical conditions by electrophysical modalities, including the application of low, medium, and high frequency electrical currents (Singh 2011). Electrotherapy reemerged as a therapy in the 1990 s, as a possible solution to the mental illness, which had once again become “brain disease” (Gilman 2008). Electrotherapy can be used in several physiological effects, namely pain relief, tissue repair, neuromuscular dysfunction, join mobility, and acute and chronic edema (Tiktinsky et al. 2010). More specifically, it is commonly applied for muscles relaxation, muscle rehabilitation and re-education through electrical muscle stimulation, prevention and retardation of disuse atrophy, improving local blood circulation, management of chronic and intractable pain, posttraumatic acute pain, postsurgical acute pain, immediate postsurgical stimulation of muscles to prevent venous thrombosis, wound healing and drug delivery (Watson 2000). Bastos et al. (2022) state that transcutaneous electrical nerve stimulation (TENS) is the most commonly used current in this type of therapy (Fig. 4). The nature of these currents allows to reduce skin impedance and promote the transport of ionic substances at very low current levels, typically below 5 mA, making the process safe as well as painless. Is important to understand that the stratum corneum, the outermost layer of the skin, acts as an effective barrier, making drug delivery into and across the skin a challenging process (Roustit et al. 2014).

**Fig. 5:**
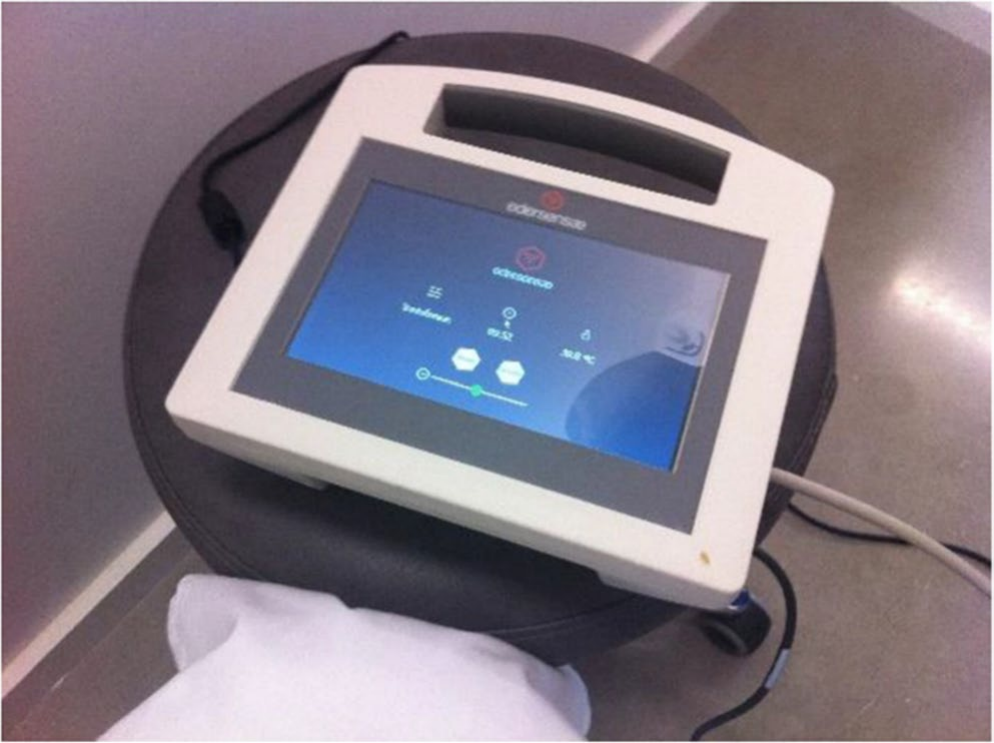
Electropelotherapy device Edersensae^®^ (Bastos et al. 2022)

To overcome this protective barrier, iontophoresis has emerged as a promising non-invasive electrotherapy method, commonly applied in physical medicine and rehabilitation for the transdermal delivery of drugs, using a low-intensity electric current in the process of transferring charged molecules (Roustit et al. 2014). Iontophoresis offers several advantages, such as faster drug release into the skin, improved passage of macro-molecules, and enhanced control over the administered dose. Pelotherapy, is also a non-invasive technique (Bastos et al. 2022). Electrotherapy has been used in the treatment of wounds, specifically to speed up the healing process (Carley and Wainapel 1985; Goldman et al. 2002; Hunckler and Mel 2017; Miguel et al. 2020; Borges et al. 2023). Several studies have explored the use of electrotherapy (Miguel et al. 2020; Borges et al. 2023) and pelotherapy (Katona et al. 2020; Almeida et al. 2023a), separately. Bastos et al. (2022) conducted the first pilot study combining these two methods to enhance treatment outcomes. This study investigates the integration of pelotherapy and iontophoresis as a unified therapeutic modality in the field of physical medicine and rehabilitation. The authors highlight that pelotherapy offers therapeutic benefits such as pain relief and anti-inflammatory effects, while iontophoresis facilitates the transdermal delivery pf ionized substances using a low-intensity electric current. Combining these modalities could enhance drug delivey by increasing skin permeability through the heat from pelotherapy, while iontophoresis drives charged molecules through the skin. However, the study also points out challenges, particularly the role of stratum corneum as a barrier, and the need for standardized quality control of peloids, which currently lack regulatory frameworks compared to pharmaceuticals. Despite these challenges, this integrated approach shows potential for improving treatment outcomes for rheumatic and dermatological conditions. Nevertheless, further clinical research is necessary to optimize treatment protocols and confirm its effectiveness. According to these authors, several experimental factors must be taken into account for transdermal delivery activation through electrical driving forces. These factors include current intensity (0.5 mA/cm^2^), temperature (40–45 °C), application duration (15–20 min), and the electrode material chosen. A device was developed (Fig. 5) to be used in electropelotherapy, specifically with maturated peloids (clays and medicinal-mineral waters), excluding those containing salty phases.

The limited evidence highlights a significant research gap in the field, underscoring the need for further investigations to evaluate the clinical applicability, underlying mechanisms, and safety of electropelotherapy, particularly in human and dermal therapeutic contexts.

## Concluding remarks

Electropelotherapy represents an innovative and relatively recent therapeutic approach with promising applications in human health, particularly in areas such as physical rehabilitation, and skin recovery treatments. Although the individual benefits of electrotherapy and pelotherapy in promoting health and well-being are well-documented, their combined use remains largely underexplored. To date, scientific literature includes only a limited number of experimental models exploring this integration, most notably a pilot study conducted in equine therapy, leaving a gap regarding its translational potential in human clinical contexts.This underscores the need for further comprehensive research to better understand the mechanisms, efficacy, and safety of this combined therapy in humans, especially in dermal applications.

## Limitations and future directions

Despite the physiological plausibility of this combined approach, particularly in enhancing skin permeability and therapeutic bioavailability, current evidence concerning the dermal bioaccessibility of peloid components remains scarce. Previous studies have predominantly focused on the thermal and rheological properties of peloids, with limited attention given to their interaction with skin barrier or their potential for ion exchange and percutaneous absorption. Additionally, it is necessary to deepen the knowledge related to the challenges associated with penetrating the stratum corneum and the resulting implications for dermal bioaccessibility, a scientific field that has been relatively underexplored so far. This knowledge gap is especially critical, as the stratum corneum represents a major obstacle to percutaneous absorption, and the electrochemical behavior of mineral ions in therapeutic muds under electric stimulation has not been adequately characterized.

To address these limitations, future research should focus on the development of well-controlled clinical trials involving human participants to evaluate the safety, therapeutic efficacy, and clinical applicability of electropelotherapy. Moreover, there is a pressing need for the standardization of peloid formulations, with clearly defined mineralogical, chemical, and microbiological profiles to ensure consistency across studies. Mechanistic investigations are also essential to explore the pathways of ion transport, skin permeability modulation, and the kinetics of bioactive compound delivery through the skin. Furthermore, toxicological assessments, ion release profillin, and in vitro skin permeation studies are essential to establish the safety parameters for clinical use. Incorporating advanced technologies capable of simultaneously delivering controlled thermal and electrical stimuli may also enhance treatment precision and reproducibility, By addressing these gaps, future research can contribute to establishing electropelotherapy as a safe, standardized, and effective modality in both dermatological and rehabilitative clinical settings. Future investigations, including those already underway, should focus on evaluating the clinical potential of combining electrotherapy and pelotherapy, particularly for skin recovery, but also on overcoming the challenges posed by the skin barrier in the treatment of dermal conditions.

Figure Routes of penetration by electric current and heat, aiming iontophoresis in conjunction with pelotherapy (Bastos et al. 2022).

## Data Availability

Data used is available on the manuscript.
